# New Alzheimer’s disease model mouse specialized for analyzing the function and toxicity of intraneuronal Amyloid β oligomers

**DOI:** 10.1038/s41598-019-53415-8

**Published:** 2019-11-22

**Authors:** Tomoyo Ochiishi, Masami Kaku, Kazuyuki Kiyosue, Motomichi Doi, Takao Urabe, Nobutaka Hattori, Hideki Shimura, Tatsuhiko Ebihara

**Affiliations:** 1 Molecular Neurobiology Research Group and DAILAB, Biomedical Research Institute (BMRI), National Institute of Advanced Industrial Science and Technology (AIST), 1-1-1, Higashi, Tsukuba Ibaraki, 305-8566 Japan; 2 Faculty of Health and Science, Uekusa Gakuen University, 1639-3, Ogura-cho, Wakaba-ku, Chiba, Chiba, 264-0007 Japan; 3 Functional Biomolecular Research Group and DAILAB, BMRI, AIST, 1-8-31, Midorigaoka, Ikeda Osaka, 563-8577 Japan; 4 Department of Neurology, Juntendo University Urayasu Hospital, 2-1-1, Tomioka, Urayasu Chiba, 279-0021 Japan; 5 Department of Neurology, Juntendo University School of Medicine, 2-1-1, Hongo, Bunkyo-ku Tokyo, 113-8421 Japan

**Keywords:** Alzheimer's disease, Experimental models of disease

## Abstract

Oligomers of intracellular amyloid β protein (Aβ) are strongly cytotoxic and play crucial roles in synaptic transmission and cognitive function in Alzheimer’s disease (AD). However, there is currently no AD model mouse in which to specifically analyze the function of Aβ oligomers only. We have now developed a novel AD model mouse, an Aβ-GFP transgenic mouse (Aβ-GFP Tg), that expresses the GFP-fused human Aβ_1-42_ protein, which forms only Aβ oligomers within neurons throughout their life. The fusion proteins are expressed mainly in the hippocampal CA1-CA2 region and cerebral cortex, and are not secreted extracellularly. The Aβ-GFP Tg mice exhibit increased tau phosphorylation, altered spine morphology, decreased expressions of the GluN2B receptor and neuroligin in synaptic regions, attenuated hippocampal long-term potentiation, and impaired object recognition memory compared with non-Tg littermates. Interestingly, these dysfunctions have already appeared in 2–3-months-old animals. The Aβ-GFP fusion protein is bioactive and highly toxic, and induces the similar synaptic dysfunctions as the naturally generated Aβ oligomer derived from postmortem AD patient brains and synthetic Aβ oligomers. Thus, Aβ-GFP Tg mouse is a new tool specialized to analyze the function of Aβ oligomers *in vivo* and to find subtle changes in synapses in early symptoms of AD.

## Introduction

Progressive memory loss and cognitive dysfunction are characteristic features of Alzheimer’s disease (AD). Disruption of the episodic memory appears to be the first cognitive disorder in AD patients and intraneuronal amyloid β protein (Aβ) oligomers might be critically involved in the onset of AD^1^. The toxicity of soluble Aβ oligomers is now definitely established by AD brain extracts or oligomers of synthetic origin. They induce decreased spine number, alter the synaptic morphology, disrupt hippocampal long-term potentiation (LTP), impair the cognitive functions, and increase phosphorylation of tau protein^2–13^. In particular, low molecular weight oligomers (8–70 kD) are much more biologically active than the higher associated oligomers^14^. However, these are all results obtained from exogenous applications of oligomers to ventricles of animals, cultured brain slices or dissociated neuronal cell cultures.

There are also many AD model mice that overexpress amyloid precursor protein (APP) with single or multiple familial AD (FAD) mutations using various promoters. However, these models reproduce only a part of AD pathology: they exhibit the extracellular deposition of Aβ and the formation of senile plaques, but some models do not show the intracellular accumulation of abnormal phosphorylated tau protein and neuronal loss. In many cases, these mice showed cognitive dysfunction prior to plaque formation^15–20^. In these models, it is very difficult to discern which pathological feature is induced by Aβ oligomers and which by fibrils. The model that overexpresses APP E693Δ might be the only one that exhibits the pathological feature of Aβ oligomers *in vivo*^21^. This model mouse exhibited no extracellular amyloid deposits but exhibited age-dependent intracellular accumulation of Aβ oligomers, abnormal tau phosphorylation, and LTP and memory impairments. However, these model mice overproduce not only the Aβ fragment but also other fragments containing the neuroprotective effects, therefore, it cannot be said to accurately represent the pathological features of AD^22–30^.

We developed a new fusion protein of human Aβ_1–42_ and GFP that enabled us to observe the GFP fluorescence even if the Aβ was aggregated^31^. Nuclear magnetic resonance (NMR), electron microscopy (EM), and fluorescence correlation spectroscopy (FCS) revealed that this fusion protein forms oligomers consisting mainly of 2 to 7 Aβ_1-42_-GFP molecules, both *in vivo* and *in vitro*. In the current study, we generated a new transgenic mouse (Aβ-GFP Tg) using the same fusion DNA fragment under the chicken β-actin promoter^31,32^. This fragment has no signal peptide to provoke the secretion of the fusion molecule from the cell, thus Aβ oligomers accumulate inside neurons in Aβ-GFP Tg mice generally. To investigate the biological activities of the Aβ-GFP fusion protein, we examined these mice using electrophysiological, behavioral, biochemical, and morphological methods and revealed that it has strong toxicity against synaptic function from young age. This suggests that Aβ-GFP fusion protein has the very similar function to that of the normal soluble Aβ oligomer that derive from the AD patients or synthetic peptide. Therefore, we have succeeded in developing the useful model mouse that specialized for investigating the function of intracellular Aβ oligomers.

## Results

### Generation of Aβ_1-42_-GFP transgenic mice

In our previous study, we have developed the new fusion protein of human Aβ_1-42_ and GFP that formed oligomer consisting of mainly 2 to 7 of Aβ_1-42_-GFP fusion molecules both *in vivo* and *in vitro*^31^. Using a DNA fragment encoding a same fusion protein of human Aβ_1-42_ and GFP, we generated a new transgenic mouse (Aβ-GFP Tg) under the control of the chicken β-actin promoter^31,32^. Three lines of Aβ-GFP Tg mouse were established. They successfully mate, show normal growth, and live for more than 2 years. For the studies reported here, we chose a single line (line 415) that showed the strongest GFP fluorescence intensity in the brain tissues.

**Figure 1:**
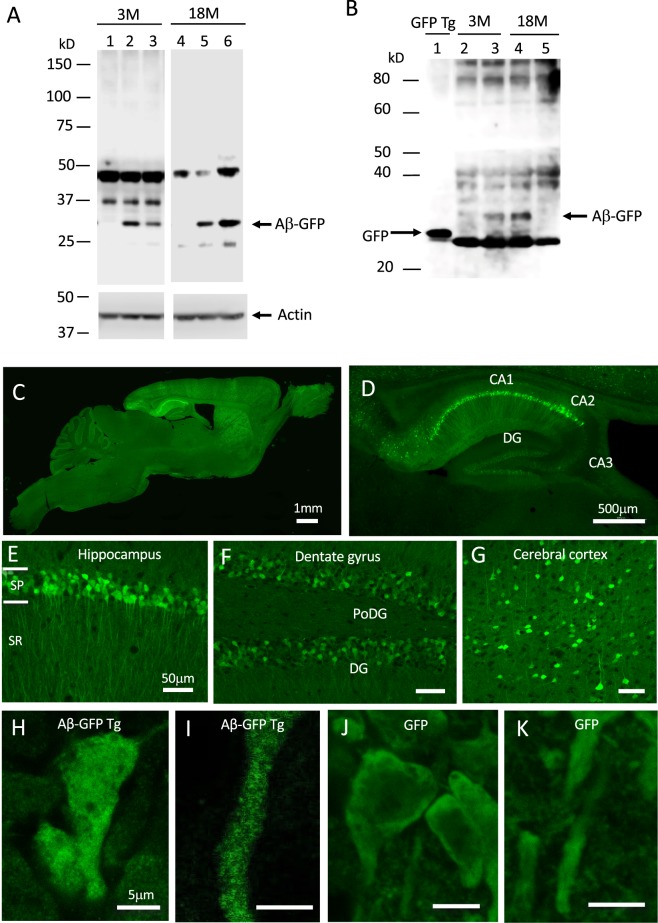
Expression of Aβ-GFP fusion protein in Aβ-GFP Tg mice brain. (**A)** Anti- Aβ (6E10) immunoblot analysis of brain homogenates (10 μg/lane) from young (3-months old; 3 M) and aged (18-months old; 18 M) Aβ-GFP Tg (lanes 2, 3, 5, 6) and non-Tg (lanes 1, 4) mice. Aβ-GFP Tg mice showed an immunopositive band around 33 kD that is the expected molecular weight of a monomer of the Aβ-GFP fusion protein. Other bands observed both from Aβ-GFP Tg and non-Tg mouse homogenates, were non-specific. (**B)** Anti-GFP immunoblot analysis. A strong single band was observed in the GFP-Tg mice (lane 1). As in (**A)**, immunopositive bands around 33 kD were observed only in the homogenate from Aβ-GFP Tg (lanes 3, 4) and not in those from the non-Tg mice (lanes 2, 5). In addition, homogenates from Aβ-GFP Tg mice also contained faint bands at the same position as the GFP Tg. (**C,D)** Sagittal sections from 12-month-old Aβ-GFP Tg mice. The Aβ-GFP fusion proteins were expressed mainly in cerebral cortex, hippocampus, thalamus, and striatum. The strongest expression was observed in the hippocampal CA1-CA2 region. (**E–G)** Magnified views of hippocampal CA1 region (**E**), dentate gyrus (**F**), and cerebral cortex (**G**). The fusion proteins were intensely expressed in CA1-CA2 pyramidal cells and their dendrites but only weakly in granule cells in dentate gyrus (DG). (**H–K)** Magnified views of CA1 pyramidal cell body and its dendrites from Aβ-GFP Tg (**H,I**) and GFP-Tg mice (**J,K**). Aβ-GFP fusion protein was distributed as small dots, although GFP alone distributed uniformly in cell body and dendrites. Scale bars; 1 mm (**C**), 500 μm (**D**), 50 μm (**E–G**) and 5 μm (**H–K**). Abbreviations, SP; stratum-pyramidale, SR; stratum-radiatum, PoDG; polymorphic layer of dentate gyrus.

Immunoblot analyses of hippocampal homogenates from 3- and 18-month-old Aβ-GFP Tg mice, using the 6E10 antibody that is specific to APP/Aβ, showed a band approximately 33 kD, consistent with the size of the monomeric Aβ-GFP fusion protein (Fig. 1A, lanes 2, 3, 5, 6, and Supplementary Fig. S1A). The level of fusion protein expression varied among individuals, but average levels were not largely different between young and aged mice. Because other sized bands were also detected in non-Tg littermate homogenates, these could be non-specific. As the GFP is fused to the C-terminal of Aβ, the Aβ-GFP fusion protein might be cleaved by secretases such as γ secretase and separated into Aβ and GFP. To check this possibility, we also conducted immunoblot analyses using anti-GFP antibody. The GFP Tg mice showed a strong band of approximately 27 kD (Fig. 1B, lane 1). In the 3- and 18-month-old Aβ-GFP Tg mice, we observed a very weak immunopositive band at the same position as the GFP Tg mice (Fig. 1B, lanes 3, 4, and Supplementary Fig. S1B), compared to much stronger bands corresponding to monomeric Aβ-GFP fusion protein (around 33 kD). These results suggested that the Aβ-GFP fusion protein in the Aβ-GFP Tg mice brain is not easily cleaved by secretases but remains as a fusion protein. Oligomer formation of Aβ-GFP fusion protein in Aβ-GFP mice brain was confirmed by the immunoprecipitation by using a GFP-Trap^®^_MA beads and western blot analyses by 6E10 antibody. Both monomer and possibly dimer bands of Aβ-GFP fusion protein were detected in Aβ-GFP Tg mice but no bands were detected in the same position in GFP Tg control (Supplementary Fig. S1C).

### Aβ_1-42_-GFP Tg mice accumulate Aβ oligomers within neurons

The expression pattern of Aβ-GFP fusion protein in the brain was observed by confocal microscopy. Intense expressions were observed in cell bodies and their dendrites in cerebral cortex, hippocampus, striatum, and thalamus and only faintly in the other brain regions we examined (Fig. 1C). The strongest expression was observed in pyramidal cells and their apical dendrites of the CA1-CA2 regions in hippocampus (Fig. 1D,E). By comparison, expression levels were relatively weak in CA3 and dentate gyrus (Fig. 1F). In cerebral cortex, Aβ-GFP-expressing cells were scattered in all cell layers (Fig. 1G). In higher magnification views of pyramidal cells in hippocampus, Aβ-GFP fluorescence was observed as many small dots throughout the cell bodies and dendrites (Fig. 1H,I), while in GFP Tg mice, GFP fluorescence was uniformly and smoothly distributed with no dot-like structures detected inside cells (Fig. 1J,K). Double labeling of Aβ-GFP Tg mice brain tissues using the 6E10 antibody and GFP antibody revealed that GFP staining and 6E10 staining completely co-localized at any parts in the cells (Supplementary Fig. S2). These results suggest that Aβ-GFP fusion proteins are polymerized as oligomers in cell bodies and dendrites.

**Figure 2:**
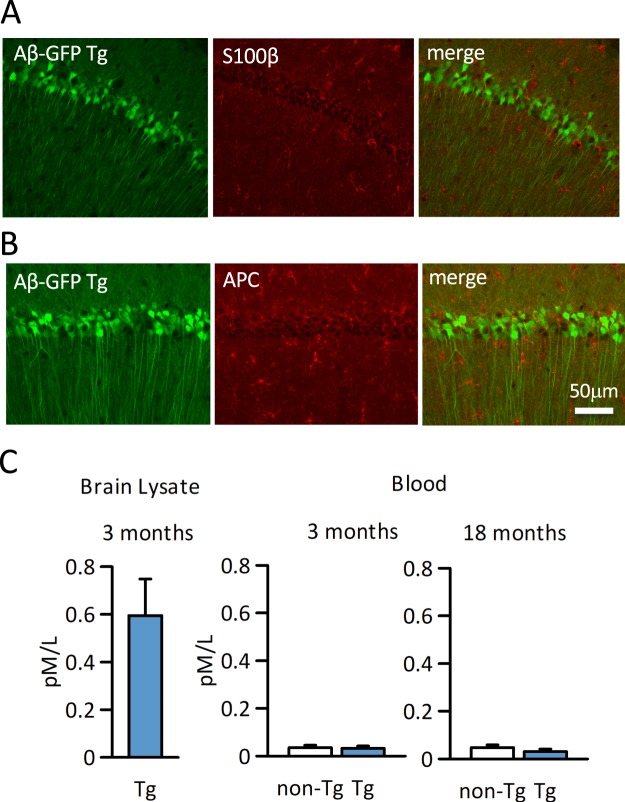
Expression of Aβ-GFP fusion protein in glial cells and blood. (**A,B)** Hippocampi from 18-months-old Aβ-GFP Tg mice were immunostained with astrocyte (S100β, **A**) and oligodendrocyte (APC, **B**) markers. As each merged image shows, Aβ-GFP fusion protein was expressed only in neurons and no double labeled cells were detected in either astrocyte or oligodendrocytes. (**C)** Concentration of Aβ_1-42_ derived from Aβ-GFP fusion protein in brain tissue and blood were measured using a human Aβ_1-42_ detection kit. At 3-month-old, human Aβ_1-42_ concentration is very high in Aβ-GFP Tg brain. However, the blood concentration is very low and did not increase even in 18-month-old Aβ-GFP Tg mice. There were no significant differences between Aβ-GFP Tg and non-Tg mice (Mann-Whitney’s U test, n = 4 animals each (serum), n = 3 animals each (brain homogenate), data are presented as means ± SEM).

Using the hippocampal tissues of aged mice, we immunohistochemically examined whether Aβ-GFP fusion protein is also expressed in glial cells. Aβ-GFP expressing cells were not labeled with either the astrocyte marker anti-S100β antibody (Fig. 2A) or with the oligodendrocyte marker anti-APC antibody (Fig. 2B), which means that the Aβ-GFP fusion protein is expressed only in the neuronal cells. Also, Aβ-GFP expressing cells were not labeled with microglia marker anti-Iba1 antibody. To examine the inflammation increases in Aβ-GFP Tg mice, we counted the number of Iba1 positive cell in hippocampus CA1 area, however, there were no significant difference between Aβ-GFP Tg and non-Tg mice (Supplementary Fig. S3). This result suggested that no extra inflammation occurred in Aβ-GFP Tg mice.

The Aβ-GFP fusion peptide does not contain the signal sequences that provoke its secretion from the cell. To confirm that the Aβ-GFP fusion protein is not secreted extracellularly, the blood concentration of human Aβ_1-42_ was measured in 3- and 18-month-old mice by ELISA. As shown in Fig. 2C, even though the high expression of human Aβ_1-42_ can be observed in the forebrain of 3-month-old Aβ-GFP Tg mice, there was no significant difference in blood concentration of Aβ in Aβ-GFP Tg and non-Tg mice. The antibody included in the kit we used is specific for human Aβ_1-42_ and the cross reactivity to the mouse Aβ is very low (about 0.5%, manufacturer’s instruction), hence the results of non-Tg mice could be non-specific, suggesting that the fusion protein is not generally secreted from neuronal cells of Aβ-GFP Tg mice.

### Aβ_1-42_-GFP Tg mice exhibit no extracellular amyloid depositions but increased phosphorylation of tau protein in brains

**Figure 3:**
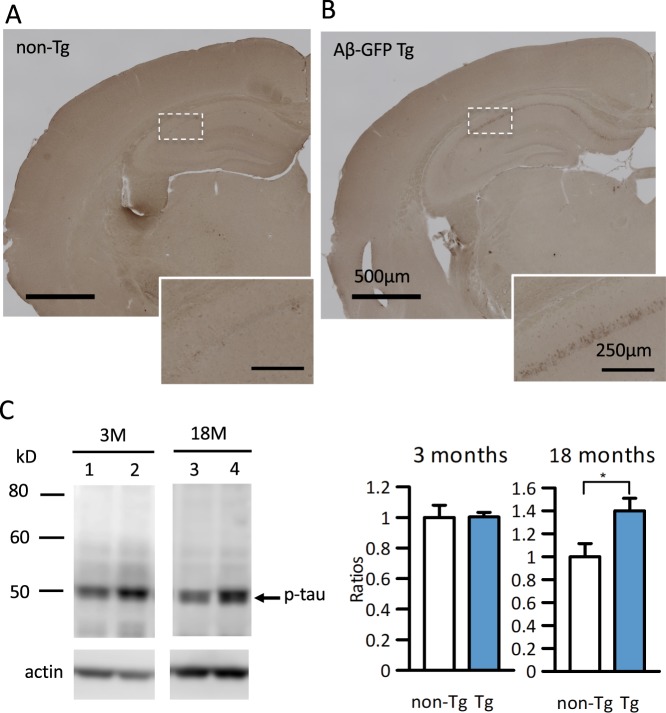
Aβ-GFP Tg mice do not exhibit the extracellular deposition of Aβ but increased phosphorylated tau. (**A,B)** Anti-Aβ (6E10) immunostaining of brain sections of 24-month-old non-Tg (**A**) and Aβ-GFP Tg mice (**B**). Insets in (**A**) and (**B**) show the magnification of the boxed areas of the hippocampal CA1 pyramidal cell layer. Although the pyramidal cells of hippocampus CA1 regions were immunopositive in Aβ-GFP Tg mice (inset in **B**), no extracellular amyloid depositions were observed in any regions we examined. No immunoreactivity was observed in non-Tg littermates (**A**). Scale bars; 500 μm (**A,B**), 250 μm (insets). (**C)** Representative immunoblots showing the expression of phosphorylated tau (p-tau) in hippocampal homogenates (3- and 18-month-old: 30 μg/lane each) in non-Tg (lanes 1, 3) and Aβ-GFP Tg (lanes 2, 4). Immunoblotting of actin served as loading control and band intensities were normalized by it. Histograms represent the ratios of phosphorylated tau in Aβ-GFP Tg against non-Tg mice. No significant difference was observed between Aβ-GFP Tg and non-Tg mice in young age but tau phosphorylation was increased significantly in Aβ-GFP Tg mice at 18-month-old (*p < 0.05, Mann-Whitney’s U test, n = 8 (3 M) and 8 (18 M) animals each, data are presented as means ± SEM).

AD is characterized by the extracellular deposition of Aβ and intracellular accumulation of hyperphosphorylated tau protein. Both of these AD pathologies were examined by immunohistochemistry and immunoblot in the Aβ-GFP Tg mice. No extracellular amyloid plaques were detected by anti-Aβ antibody (6E10) immunostaining at 24- month-old mice (Fig. 3B) in any regions we examined, even though abundant intracellular immunoreactivities were detected in cell bodies of the CA1-CA2 regions of hippocampus (Fig. 3B, inset). Non-Tg littermates exhibited no 6E10 antibody immunoreactivity (Fig. 3A). No extracellular plaque formation was confirmed by the methoxy-X04 staining, which specifically recognizes amyloid plaques in brain tissues (Supplementary Fig. S4A). Also, we tried to see the extra fibril formation in aged Aβ-GFP Tg mice by dot blot analyses using specific antibody for amyloid fibrils. The average expression level of fibrils in Aβ-GFP Tg mice was similar with that of non-Tg mice (Supplementary Fig. S4B). Based on our finding, we surmise that because the Aβ-GFP fusion protein is not secreted extracellularly, or even if it is secreted, these fusion proteins form only oligomers^31^, it may be impossible to form plaques.

Aβ oligomers have been demonstrated to cause the hyperphosphorylations of tau protein *in vivo*^2,3,33^ and *in vitro*^21^. Therefore, we examined the phosphorylation of tau in Aβ-GFP Tg mice brains. Immunoblot analyses of hippocampal homogenates from 3- and 18-month-old mice were performed with the phospho-T231 antibody, which specifically recognizes phosphorylated tau protein. We quantified phosphorylated tau by measuring the densities of blot bands and normalizing them by actin, and then calculated the ratios against non-Tg mice. Phosphorylation ratios of Aβ-GFP Tg mice were almost same in non-Tg mice in young age but were significantly increased in aged Aβ-GFP Tg mice (Fig. 3C, Supplementary Fig. S5).

**Figure 4:**
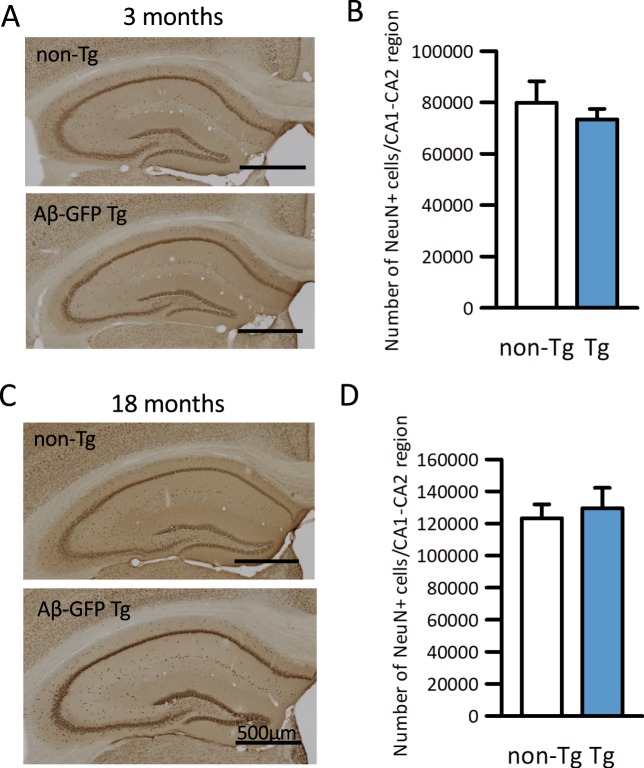
Aβ-GFP fusion protein did not induce neuronal cell death in hippocampus. (**A,C)** Anti-NeuN immunostaining of coronal sections of Aβ-GFP Tg and non-Tg littermates at 3- month (**A**) and 18- month (**C**) old. (**B,D)** Total number of NeuN-positive cells in the CA1-CA2 region of hippocampus measured using Stereo Investigator. Histograms show that there were no significant differences in the numbers of NeuN-positive cells between Aβ-GFP Tg and non-Tg in either 3-month (**B**) or 18-month (**D**) old mice (Mann-Whitney’s U test, n = 7 animals each, data are presented as means ± SEM). Scale bars; 500 μm.

Then, to determine whether neuronal loss occurred in Aβ-GFP Tg mice, brains from 3- and 18-24-month-old mice were immunostained with anti-NeuN antibody, which stains the entire nuclei of mature neurons, and counted the total numbers of immunopositive cells in the hippocampal CA1-CA2 regions using stereology microscope. At both ages, the total numbers of immunopositive cells in the Aβ-GFP Tg mice were not significantly different than in their non-Tg littermates (Fig. 4), indicating that cell death rates were normal in the Aβ-GFP Tg mice.

### Electrophysiological analysis

**Figure 5:**
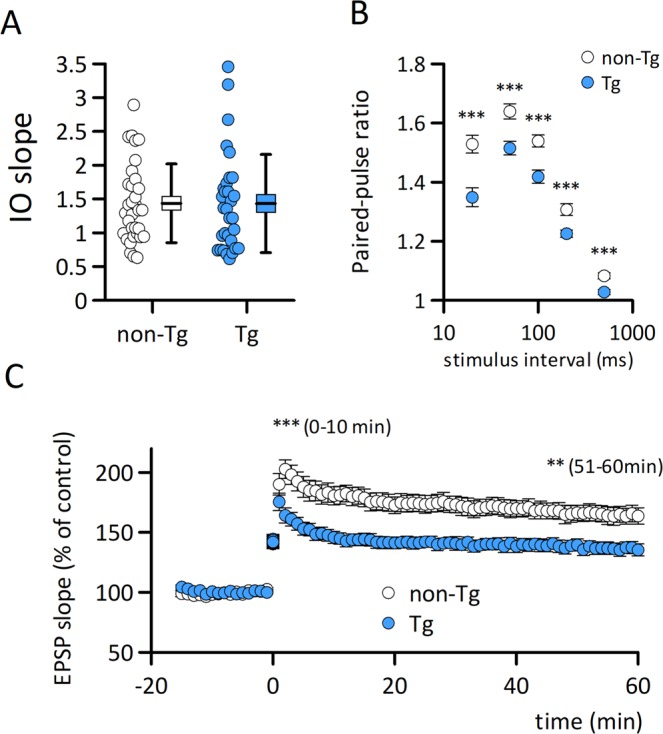
Aβ-GFP Tg mice exhibit the impairment in long-term potentiation. (**A)** There are no significant difference in the slopes of the input-output relationship curve in the Schaffer collateral - CA1 synapses in Aβ-GFP Tg (n = 32 slices from 7 mice) and non-Tg (n = 33 slices from 7 mice) mice at 2-month-old. (**B)** Paired-pulse ratios of CA1 synapse fEPSPs in Aβ-GFP Tg (n = 32–33 slices from 7 mice) and non-Tg (n = 32–33 slices from 7 mice) mice at 2-month-old. Paired-pulse facilitation was negatively affected in the Aβ-GFP Tg mouse slices (***p = 0.0001 at 20 ms; p = 0.0006 at 50 ms; p = 0.0002 at 100 ms; p = 0.0009 at 200 ms; p = 0.0002 at 500 ms). (**C**) Theta burst stimulation induced LTP was impaired in Aβ-GFP Tg (n = 19 slices from 6 mice) compared to non-Tg (n = 16 slices from 7 mice, p < 0.001) mouse slices.

To determine whether synaptic function was altered in the model mice, we examined field EPSPs in the CA1 striatum radium in acute hippocampal slices from both Aβ-GFP Tg and non-Tg mice. Stimulus response curves from Aβ-GFP Tg and non-Tg littermates were generated to evaluate input-output relationships in the Schaffer-collateral - CA1 synapses. There were no significant differences in the slopes of the input-output curves between the two (Fig. 5A, non-Tg: mean slope 1.44 ± 0.10, n = 33, Aβ-GFP Tg: 1.43 ± 0.14, n = 32).

To assess short-term synaptic plasticity, we conducted a paired-pulse stimulus protocol. Paired-pulse facilitations were observed in both non-Tg and Aβ-GFP Tg mice at stimulus intervals of 20 ms, 50 ms, 100 ms, 200 ms and 500 ms; however, slices from Aβ-GFP Tg mice showed significantly less facilitation than those from non-Tg mice at all intervals tested (Fig. 5B).

LTP is a well characterized form of synaptic plasticity and is considered the cellular event for memory formation and storage. We applied theta-burst stimulation to induce LTP and compared the synaptic changes in slices from Aβ-GFP Tg mice and their non-Tg littermates. Both the induction and maintenance of LTP were impaired in Aβ-GFP Tg mouse slices beginning 10 min (non-Tg: 188.63 ± 7.23%, n = 16 slices from 7 mice, Aβ-GFP Tg: 154.66 ± 4.88%, n = 19 slices from 6 mice) and continuing 60 min (non-Tg: 164.98 ± 6.87%, n = 16 slices from 7 mice, Aβ-GFP Tg: 135.80 ± 4.66%, n = 19 slices from 6 mice) after induction (Fig. 5C), suggesting that Aβ-GFP Tg mice presented both presynaptic and postsynaptic malfunctions.

### Morphological and biochemical changes in Aβ-GFP Tg mice synapses

**Figure 6:**
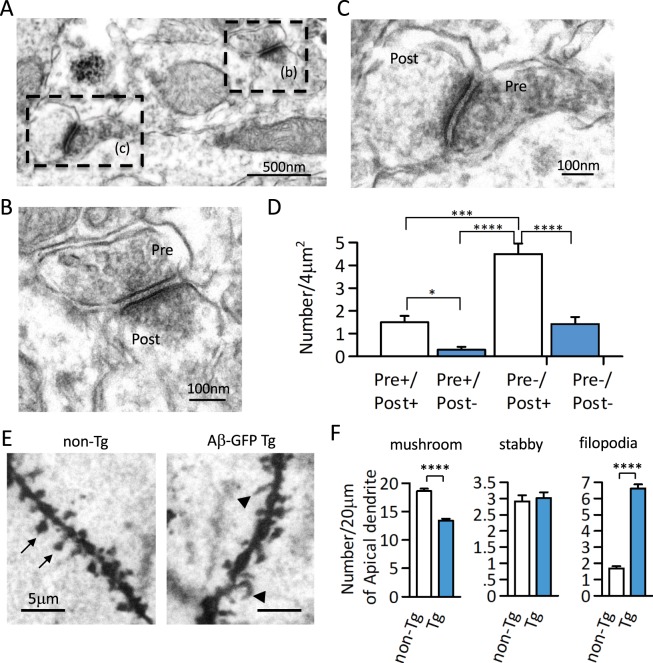
Synaptic expression of Aβ-GFP fusion protein and morphological changes in synapses in Aβ-GFP Tg mice. (**A–C)** Representative Immuno-EM image by anti-GFP antibody in Aβ-GFP Tg mice hippocampus at 3-month-old. The dotted rectangle in (**A**) are shown at higher magnification in (**B**) and (**C**). (**D**) Histograms representing the numbers of immunopositive synaptic structures per unit areas of stratum-radiatum in the hippocampal CA1 region. Aβ-GFP fusion protein was expressed mainly in the post synaptic sites (*p < 0.05, ***p < 0.0050, ****p < 0.0001 by one way ANOVA, n = 24 areas, data are presented as means ± SEM). (**E**) Golgi staining of apical dendrite from non-Tg and Aβ-GFP Tg mice hippocampal CA1 regions. Arrows showed spine and arrow heads showed filopodia. Compared to the non-Tg, Aβ-GFP Tg mice had significantly fewer spines and significantly more filopodia (****p < 0.0001 by Student’s t-test, n = 5 animals each, 20 μm length of dendrites from 24–27 areas each, data are presented as means ± SEM).

As the electrophysiological analyses suggested the possibility of synaptic changes in Aβ-GFP Tg mice, we examined the expression of Aβ-GFP fusion protein in synapses using immuno-EM. Figure 6A–C show the typical anit-GFP antibody staining pattern of Aβ-GFP fusion protein in the synaptic region of hippocampal CA1. To quantitate these observations, we counted the immunopositive synaptic structures per unit areas of stratum-radiatum in hippocampal CA1. Aβ-GFP fusion protein was predominantly expressed at postsynaptic sites and less frequently at presynaptic sites (Fig. 6D).

To see whether this expression affected synapse structures, we next examined the morphology of dendritic protrusions on apical dendrites of hippocampal CA1 in 3-month-old Aβ-GFP Tg and non-Tg mice using Golgi staining (Fig. 6E). Although total number of protrusions were approximately the same in the Aβ-GFP Tg and non-Tg mice, the shapes of protrusions were slightly altered in the Aβ-GFP Tg mice. Almost all the protrusions in non-Tg mice were mushroom or stubby type spines and the frequency of filopodia-like structures was very low. However, in the Aβ-GFP Tg mice, the mushroom types spines were much fewer, and the number of filopodia-like structures was significantly increased compared to the non-Tg (Fig. 6F).

**Figure 7:**
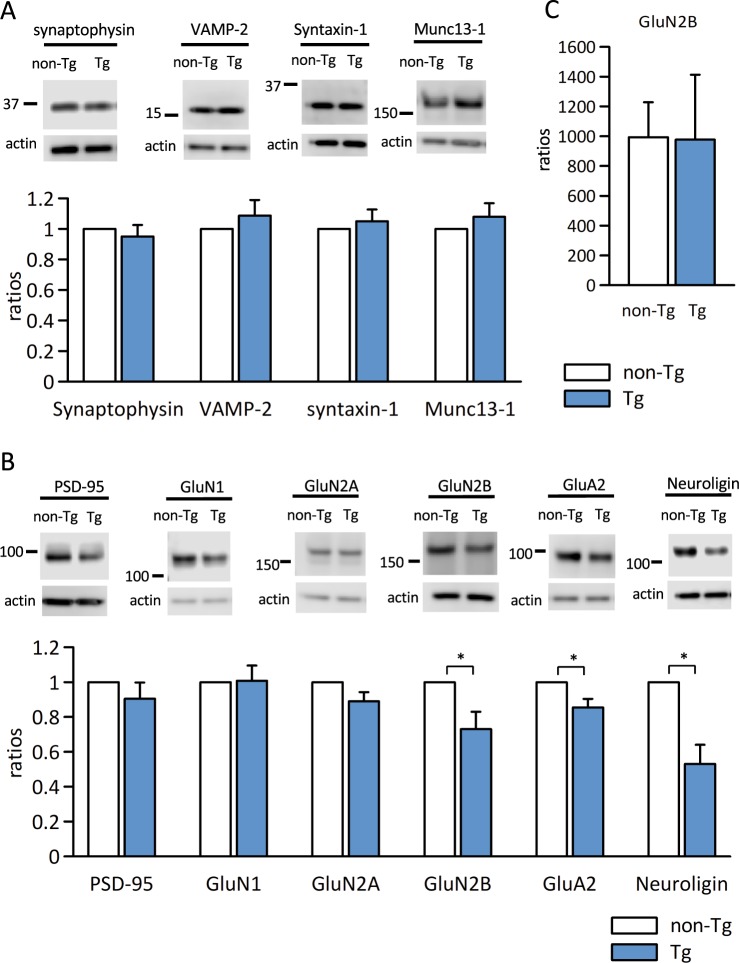
Immunoblot analysis of expression ratios of the pre and postsynaptic proteins in Aβ-GFP Tg and non-Tg mice synapse. (**A,B**) Synaptosome fractions from hippocampus (20 μg/lane) were immunoblotted against presynaptic markers (**A**; synaptophysin, VAMP-2, syntaxin-1, and Munc13–1) and postsynaptic markers (**B**; PSD-95, GluN1, GluN2A, GluN2B, GluA2, and neuroligin). We prepared 4 sets of Aβ-GFP Tg/non-Tg samples. Band intensities of Aβ-GFP Tg mice were normalized by those of actin and the expression were calculated ratios with the values of non-Tg mice as 1. There was no difference in expression ratios of the presynaptic proteins we examined. However, expression of the postsynaptic proteins GluN2B, Glu2A, and neuroligin were significantly decreased in Aβ-GFP Tg mice at 3-month-old (*p < 0.05 by Mann-Whitney’s U test, n = 4 that included hippocampi from 3 animals each, data are presented as means ± SEM). (**C**) The expression ratios of GluN2B in homogenate. There was no significant difference in the expression ratios of GluN2B in homogenates of Aβ-GFP Tg and non-Tg mice (Mann-Whitney’s U test, n = 5 animals each, data are presented as means ± SEM).

To understand the molecular alterations in Aβ-GFP Tg mice synapses, we next examined the expression levels of several pre and postsynaptic proteins that are related to the synaptic transmission. There were no significant differences in the expressions of any of the presynaptic proteins we examined (synaptophysin, VAMP-2, syntaxin-1, and Muc13-1) in the Aβ-GFP Tg compared with non-Tg littermate mice (Fig. 7A, Supplementary Figs. S6A, S7A). However, of the postsynaptic proteins we examined (PSD-95, GluN1, GluN2A, GluN2B, GluA2, and neuroligin), the expressions of GluN2B, GluA2, and neuroligin were significantly decreased compared to their levels in non-Tg mice (Fig. 7B, Supplementary Figs. S6B, S7B). Snyder *et al*.^34^ reported that Aβ reduced synaptic GluN2B receptors by promoting endocytosis of these receptors. Therefore, we used hippocampal homogenates to examine the total expression of GluN2B (Fig. 7C). The results indicated that the expression level of GluN2B in Aβ-GFP Tg mice was same as in non-Tg mice in homogenates. These results suggested that Aβ-GFP fusion protein induced the reduction of synaptic GluN2B receptors and increased the extrasynaptic ones in Aβ-GFP Tg mice.

### Aβ-GFP Tg mice show impaired recognition memory

**Figure 8:**
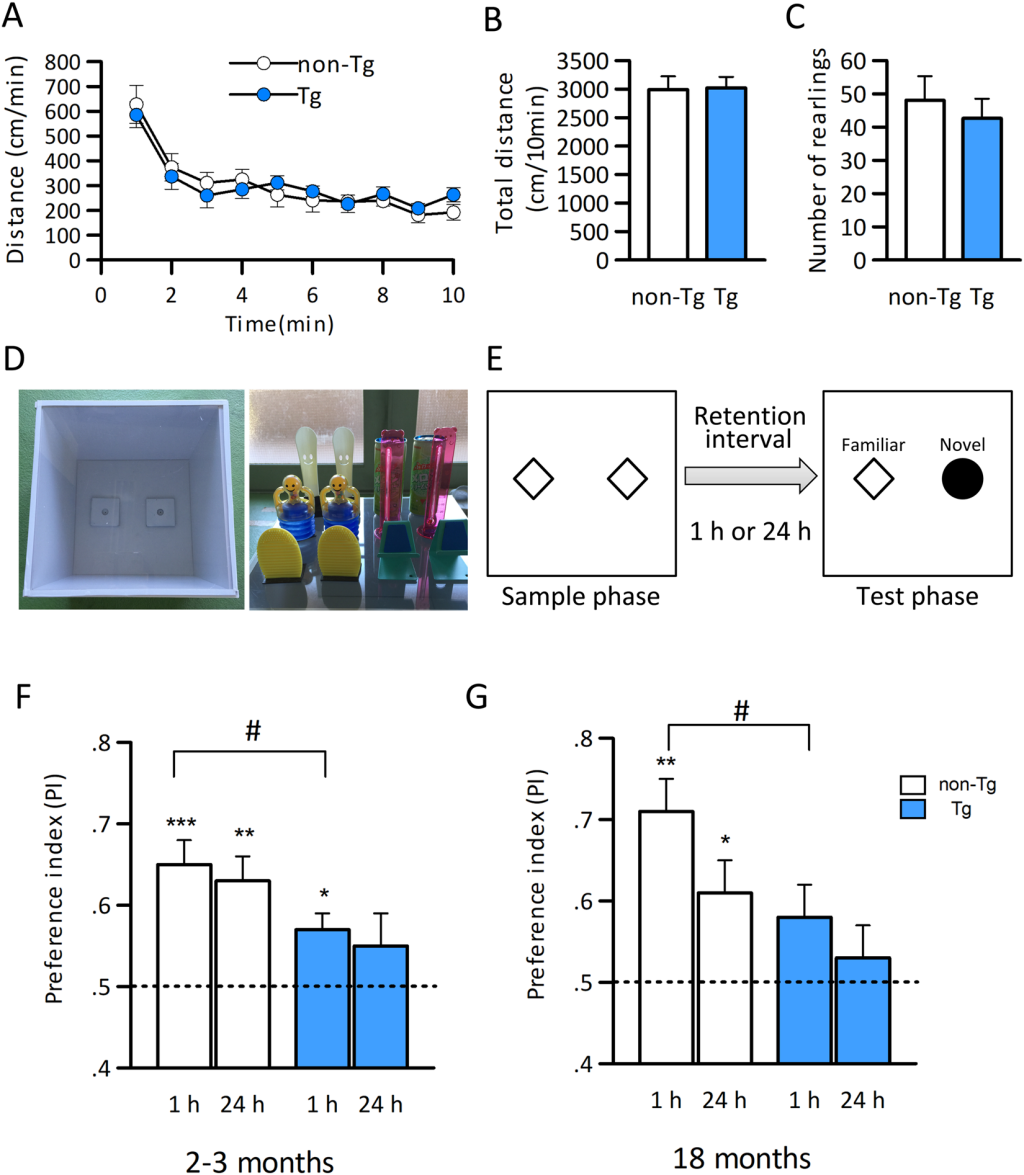
Aβ-GFP Tg mice exhibit the memory impairment both in young (3 months) and aged (18 months) mice. (**A–C)**: Exploratory behavior in the open-field was measured in 18- month-old by locomotor activities (**A,B**) and the total number of rearings (**C**) over 10 min. Data are expressed as means ± SEM. (n = 11 each). There were no impairments in the OFT, indicating that exploratory behavior and locomotor activity were intact in aged Aβ-GFP Tg mice.(**D,E)**: Apparatuses and the objects used for the NORT (**D**) and schematic representation of sample and test phase conditions in the NORT (**E**). (**F,G)**: Impairment of object recognition memory in young (2–3 months) (**F**) and aged (18 months) (**G**) Aβ-GFP Tg mice. Dotted lines indicate chance performance (0.5). Data are presented as means ± SEM. ^#^p < 0.05; unpaired *t*-test to evaluate difference between Aβ-GFP Tg and non-Tg mice, ***p < 0.001, **p < 0.01, * p < 0.05; one-sample *t*-test for the PI compared with chance performance (test value = 0.5).

The electrophysiological, morphological, and biochemical analyses suggest that Aβ-GFP Tg mice would exhibit memory impairments. Therefore, we examined cognitive function in Aβ-GFP Tg mice. Firstly, to examine whether the spontaneous activities were altered in Aβ-GFP Tg mice, we conducted open field tests (OFT) and compared their general locomotion and exploratory behavior in a novel environment to those of the non-Tg mice. Open field exploratory behavior in 18-month-old mice was measured by the total distance moved and the total number of rearings over a 10 min period (Fig. 8A–C). When the session was divided into 1 min time bins, both Aβ-GFP Tg and non-Tg mice performed similarly in each of the segments (Fig. 8A, Two-way repeated measures ANOVA with GROUP (Aβ-GFP Tg and non-Tg); F_1,180_ = 0.0082, p = 0.929, and with Time (10 min) and GROUP; F_9,180_ = 0.775, p = 0.64). There were also no significant differences in the total distance or the total number of rearings (Fig. 8B,C), indicating that general locomotor activity and exploratory behavior was intact in Aβ-GFP Tg at 18-month-old.

Next, we examined the cognitive function of 2–3- and 18-month-old mice in the novel object recognition test (NORT, Fig. 8D,E). NORT is based on the spontaneous tendency of rodents to exhibit more preference for a novel object rather than for a familiar object. Initially, we compared the preference index (PI) of non-Tg mice with that of Aβ-GFP Tg mice. Mice without memory impairment spend a longer time investigating for the novel object, resulting in a higher PI. Over a short retention interval (1 h), both 2–3- and 18-month-old Aβ-GFP Tg mice exhibited significantly lower PIs than those of age-matched non-Tg mice, suggesting an impaired recognition memory in Aβ-GFP Tg mice (Fig. 8F,G). However, over a long retention interval (24 h), there were no significant differences between Aβ-GFP Tg and non-Tg mice at either 2–3- and 18-month-old. To further investigate whether memory impairment actually occurred in Aβ-GFP Tg mice after the 24 h retention interval, we also analyzed whether each of the PIs was significantly different from the chance performance (test value = 0.5, reflected no preference in exploration of any object). The PIs of the non-Tg mice were significantly different from chance performance at both 2–3- and 18-month-old. By contrast, the PIs of the Aβ-GFP Tg mice at the 24 h retention interval were not significantly different from chance performance at both 2–3- and 18-month-old, indicating that memory impairment had occurred in the Aβ-GFP Tg mice even at the 24 h retention interval.

The short retention interval at 2–3-months-old was significantly different from chance performance but those at the 18-month-old mice was not. These results indicated that the older Tg mice have an increased memory deficit when compared to the young Tg mice. Taken together, these results suggested that both young and old Aβ-GFP Tg mice exhibit impaired recognition memory compared with non-Tg mice and memory deficit were increased in older Aβ-GFP Tg mice.

## Discussion

We developed a new mouse model of AD that expresses a fusion protein of human Aβ_1-42_ and GFP. This mouse exhibited intracellular accumulation of Aβ oligomers, increased tau phosphorylation, altered spine morphology, attenuated LTP, and memory impairment in young age, but no plaque formation, no atrophy, and no neuronal cell death. Many different AD mice models have been developed, mainly using APP with single or multiple FAD mutations^15–20^. Because the cleaved APP includes not only toxic fragments like Aβ but also the neuroprotective ones like sAPPα a soluble cleavage product of APP, over expression of APP raises the risk of artificial consequences^22–30^. Thus, APP knock-in mice with FAD mutations, in which it is possible to observe the effects of Aβ at normal expression levels, has attracted attention^35^. In this study, we used only Aβ_1-42_ fused with GFP, however, it also provides the possibilities of producing fragments of Aβ alone, P3, P3-GFP, and GFP cleaved by α-secretase and γ-secretase. Immunoblot analysis of anti-GFP antibody (Fig. 1B) revealed that a very small amount of GFP fragment was produced, but nearly all of the overproduced proteins were the fusion proteins of Aβ and GFP. The P3 fraction is reported to be devoid of synaptotoxic effects and does not assemble into the soluble Aβ oligomers^36,37^, suggesting that the P3 and P3-GFP fragments do not disturb the effects of Aβ-GFP fusion protein even if they do exist. Therefore, the Aβ-GFP Tg mice should enable one to observe only the effects of Aβ-GFP fusion protein and small amounts of overproduced Aβ, without unexpected effects of other overproduced fragments.

Our previous NMR analyses demonstrated that under the polymerization conditions the aggregation processes of the Aβ-GFP fusion protein stops before fibril formation occurs. EM analyses shows that Aβ oligomers containing 2–7 fusion molecules form *in vitro*. Furthermore, FCS analyses *in vivo* showed that approximately 3 fusion molecules were polymerized in the cytoplasm of living COS7 cells^31^. In the present study, immunoblot analyses of Aβ-GFP Tg mouse brain homogenates exhibited a specific band centered at 33 kD (Fig. 1A), which correlates with the speculated molecular weight of the monomer Aβ-GFP fusion protein, and no other Aβ-GFP Tg-specific bands were observed compared to the non-Tg littermates. These findings suggest that the Aβ-GFP oligomers are SDS labile and easy to disaggregate into Aβ-GFP monomers upon denaturing by SDS. The low molecular weight oligomers extracted from AD patient brains are also easy to disaggregate into monomer or dimers by SDS^38^. Therefore, by fusing the Aβ to GFP, the Aβ-GFP Tg mice express small size Aβ oligomers consisting of perhaps only several molecules.

Detailed examinations of the relationship between the sizes and its toxicity of oligomers using the extracts of soluble Aβ from AD patients indicated that low molecular weight oligomers (8–70 kD) are more toxic than higher molecular weight ones (>70 kD) even though the high molecular weight oligomers are predominant in AD patient brains^14,38^. Taking these reports into consideration, the Aβ-GFP fusion protein should have been strongly toxic. However, because the GFP moiety is huge compared to the Aβ fragment, we considered that the function of the Aβ oligomer might be disrupted by the GFP. Therefore, we tried to confirm the toxicity of Aβ-GFP fusion proteins in neurons. Immunoblot analyses demonstrated increased levels of phosphorylated tau protein in aged mice (Fig. 3C). Unfortunately, we tried but were unable to successfully immunostain phosphorylated tau protein in neuronal cell bodies (data not shown), and did not detect neuronal cell death in either young or old animals (Fig. 4). These results may suggest that tau phosphorylation in Aβ-GFP Tg mice is not strong enough to cause neuronal cell death.

Our Aβ-GFP Tg mice exhibited impaired LTP (Fig. 5C) and reduced levels of the GluN2B receptor subunit in hippocampal synaptosomal fractions (Fig. 7B). These facts are consistent with previous data showing that LTP in the Schaffer-collateral synapses is NMDA receptor dependent^39–41^. On the other hand, the levels of GluN2B in hippocampal homogenates from Aβ-GFP Tg were the same as those from non-Tg (Fig. 7C). These results might be indicating that the Aβ-GFP fusion protein induces an increase of extrasynaptic NMDA receptors (eNMDAR) containing GluN2B subunit, although further experiments were needed to show this. Snyder *et al*.^34^ reported that applications of Aβ peptide to cultured neurons increase ratios of eNMDARs by enhancing the endocytosis of synaptic NMDAR from synapse membranes and transferring them to extrasynaptic sites. Because reductions in LTP resulting from applications of Aβ oligomers isolated from postmortem AD patients or of culture medium of an AD cell line can be rescued by a selective GluN2 inhibitor or eNMDAR antagonist memantine, the activation of eNMDARs containing GluN2B subunit may be a cause of the inhibition of LTP by Aβ oligomers^10,42^. Although these results were obtained by administrating Aβ oligomers from outside the tissue, Aβ-GFP fusion protein expressed inside neurons might have a similar effect to that of Aβ oligomers from AD patients and from cultured AD cells.

Although the expression of Aβ-GFP fusion protein is predominantly in postsynaptic sites of CA1 neurons, we also found presynaptic impairments evident as reduced paired-pulse facilitation. One possible explanation is that the Aβ-GFP fusion protein expressed in CA3 neurons may be transported to axon terminals and somehow affects the release of synaptic vesicles, although the level of fusion protein was lower than that in CA1 neurons (Fig. 1D). The other possibility is that Aβ-GFP fusion proteins at postsynaptic sites might indirectly affect the presynaptic release machinery. Neuroligin mediated signal transduction is one possible molecular candidate for postsynaptic malfunctions resulting from increased Aβ-GFP fusion protein. Neuroligin is a postsynaptic adhesion molecules and link to neurexin at presynaptic terminals. Neuroligin-neurexin interaction is involved in many functions at synapses, including synaptogenesis, maturation, stability, and plasticity^43,44^, and break down of this interaction alters paired-pulse response (PPR) in hippocampus^45^. The reduced expression of neuroligin in the synaptosome fraction of Aβ-GFP Tg mice (Fig. 7B) might have been responsible for the reduction in PPR we observed (Fig. 5B). Moreover, taking into consideration the reduced neuroligin and morphological alteration of spine and filopodia-like structures (Fig. 6E,F) in Aβ-GFP Tg mice, involvement of the neuroligin-mediated signaling pathway may also be responsible for the memory impairments observed in Aβ-GFP Tg mice.

Aβ-GFP Tg mice exhibited normal locomotive activities. Other studies have reported inconsistent activities in OFT of different transgenic AD model mice: hypoactivity in APP^SWE^/PS-1^δE9^ mice and hyperactivity in APP^SWE/^PS-1^M146L^ mice^46^. The exploratory behavior/locomotor activity in older Aβ-GFP Tg mice did not differ from that of non-Tg mice supports the idea that Aβ-GFP Tg mice can be used to evaluate pure memory impairment as an aged model of AD with intact locomotor activities. Moreover, short- and long-term memory impairments were observed both at 2–3-months and at 18-months old. In other APP Tg mice, for example APP-KI, 3xTg, and 5xFAD mice, memory impairments usually appeared at least 4 months of age or later^16,21,35,47,48^. In 3xTg mice, cognitive impairment was rescued by the clearance of intraneuronal Aβ by the intracerebroventricular injection of anti-Aβ antibody^47^. These results indicated that intracellular Aβ oligomer has a very strong toxicity against memory formation.

In conclusion, many well-known effects of Aβ oligomers can be observed in our Aβ-GFP Tg mice which abundantly express an Aβ-GFP fusion protein inside neurons. This increased intracellular Aβ-GFP oligomer resulted in serious synaptic dysfunctions and memory impairment at a very early age, even though no AD typical pathology had appeared. These results suggest that Aβ-GFP Tg mice are unique for investigating the function and toxicity of small Aβ oligomers inside neurons and might be a good model of very early stage of AD.

## Methods

### Generation of the transgenic mouse line carrying the Aβ_1-42_-GFP sequence together with the β-actin promoter

Transgenic mice expressing human Aβ_1-42_ fused with GFP were generated using a modified pEGFP-N1 vector (Takara BIO Inc, Shiga, Japan) containing a chicken β-actin promoter (pAct-Aβ_1-42_-GFP) as described previously^31,32^. Fertilized eggs from B6C3F1(C57BL/6 N × C3H/HeN) mice received microinjections of the pAct-Aβ_1-42_-GFP. Three independent stable transgenic lines were obtained and one of them was used in this study. All mice used were males and heterozygous for the transgene. Transgene expression was screened by tail PCR. The B6C3F1 strain exhibited the retinal degeneration phenotype. C57BL/6N has the *rd8* mutation which is a single nucleotide deletion in *crb1* gene and exhibits a progressive light-colored spot in the fundus of the eye already at 5 weeks of age^49,50^. On the other hand, C3H is the carrier of the *rd1* mutation, which induces early onset degeneration of photoreceptors and retinal morphology, and becomes completely blind^49,51^. Both *rd1* and *rd8* exhibit their phenotypes homozygously. Therefore, no carriers and heterozygous of these mutations were used in this experiment.

All experimental procedures performed on mice were carried out in accordance with the approved guidelines in ethical permit approved by the Institutional Animal Care and Use Committee of the National Institute of Advanced Industrial Science and Technology (Permission No. 2018-143) and in accordance with the Law No.105 passed by and the Notification No. 6 released by the Japanese Government.

### Immunohistochemistry

Immunostainings were performed as described previously^52^ with some modifications. Aβ-GFP Tg and non-Tg littermates approximately 3, 12 and 18–24 months of age were used. Briefly, animals were deeply anesthetized by isoflurane (FUJIFILM Wako Pure Chemical Corporation, Osaka, Japan) and perfused transcardially with 4% paraformaldehyde. Sagittal and coronal sections (40μm) were incubated with astrocyte marker S-100β (1:500, polyclonal, Sigma-Aldrich, MO, USA) or oligodendrocyte marker APC (1:500, monoclonal, Merc, Darmstadt, Germany) overnight at 4 °C and visualized with an Alexa 568-conjugated secondary antibodies (Molecular Probes, OR, USA). Nuclei were labeled with DAPI (Vector Laboratories, Inc., CA, USA). Low magnification images (1920 × 1440 pixels) were taken with a BZ-X800 fluorescent microscope (KEYENCE corporation, Osaka, Japan). High magnification images were taken with an Olympus FluoView 1200 (1020 × 1020 pixels, Olympus, Tokyo, Japan) confocal laser scanning microscopes. The 3,3′-diaminobenzidine (DAB) staining was performed with anti-Aβ antibody (6E10, 1:800, monoclonal, Covance Research Products Inc, NJ, USA) and NeuN antibody (1:1000, monoclonal, Merc). For 6E10 staining, tissues were pretreated with Target Retrieval Solution (Agilent Technologies Inc., CA, USA) according to the manufacturer’s instructions. After the incubation with primary antibodies, sections were treated with biotinylated goat anti-mouse IgG and avidin-biotin-peroxidase complex. Immunoreactivity was visualized by DAB using the Metal Enhanced DAB Substrate kit (Thermo Scientific, IL, USA). After dehydration and clearing in Hemo-De (Electron Microscopy Science, PA, USA), images were taken with a BZ-X800 microscope.

### Measurement of the concentration of human Aβ_1-42_ from Aβ-GFP Tg mice brain tissues and blood

The blood of 3- and 18-months-old Aβ-GFP Tg and non-Tg littermate mice (n = 4 each) were collected through the postcava using 26 gage needles under the deep anesthesia with isoflurane. Forebrain homogenates of 3-month-Aβ-GFP Tg mice were prepared as described previously^53^ (n = 3). Each serum and homogenate of forebrain from 3-month-old was measured by ELISA using a Human β Amyloid (1–42) ELISA kit *Wako*, High- Sensitive (FUJIFILM Wako Pure Chemical Corporation).

### Electron microscopic examination

Tissue samples were prepared as described previously^54^ with the following modifications. Sections (100 μm) from 3-month-old Aβ-GFP Tg mice (n = 3) were immunostainined with anti-GFP antibody (1:500, polyclonal, MBL International, MA, USA). After the DAB reaction, sections were postfixed with 1% OsO_4_, dehydrated in ethanol and propylene oxide, and embedded in an Epon812 (TAAB Laboratories Equipment Ltd, Berkshire, England). Ultrathin sections were counterstained with a 2% uranyl acetate/lead citrate solution. Observations were made with an electron microscope (H-7600, Hitachi Ltd, Tokyo, Japan) at an acceleration voltage of 80 kV.

### Cell number count

Coronal sections from 3- and 18-month-old Aβ-GFP Tg and non-Tg littermate mice were immunostained with an anti-NeuN antibody as described above. The total cell numbers of pyramidal cells in hippocampal CA1 and CA2 regions were counted using Stereo Investigator (MBF Bioscience, VT, USA).

### Golgi-Cox staining and measurement of spine and filopodia numbers

Golgi-Cox staining of coronal brain sections (100 μm) from 3-month-old Aβ-GFP Tg and non-Tg littermate mice was performed using the FD Rapid GolgiStain^TM^ Kit (FD NeuroTechnologies, Inc., MD, USA) according to the manufacturer’s instructions.

Each image was obtained under a BZ-X800 microscope with Z-series of 100–160 images taken at 0.4 μm depth intervals. Maximum projection of the Z-stack image was flattened into a single image. Using these images, the numbers of protrusions were counted in 20 μm-length dendrites from 24–27 areas in the hippocampal CA1 region in each mouse (n = 5 each) using BZ-H4M software (Keyence). The protrusions were classified as dendritic spines (mushroom, 5 μm ≥ length and ratio of neck width/head width ≥ 1.5; stubby, 1 < the length ≤ 5 μm and ratio of neck width/head width < 1.5) and filopodia-like structures (1.5 μm < length and no head)^55,56^.

### Immunoblot analysis and quantification

Four sets of synaptosome fraction samples from 3-month-old Aβ-GFP Tg and non-Tg mice were prepared as described previously^53^. Each set was a mixture of 3 individual Aβ-GFP Tg or non-Tg littermate hippocampi (totally 12 mice were used both in Aβ-GFP Tg or non-Tg mice). Proteins were subjected to SDS-PAGE and transferred to PVDF membranes. VAMP-2 (1:5000, Abcam, Cambridge, UK), Munc13-1(1:1000, Synaptic Systems, Gottingen, Germany), syntaxin-1 (1:1000, Merck), synaptophysin (1:2000, Sigma-Aldrich), PSD-95 (1:200, Merck), GluN1 (1:200, Merck), GluN2A (1:500, Merck), GluN2B (1:200, Merck), GluA2 (1:200, Merck), neuroligin 1(1:500, Synaptic Systems), and actin (1:2000, Merck) antibodies were used to probe the corresponding proteins and ECL was used for detection (Immunostar Zeta and Immunostar LD, FUJIFILM Wako Pure Chemical Corporation).

Immunoblotting of homogenates of individual Aβ-GFP Tg and non-Tg mouse hippocampi was performed using 6E10, GFP (1:200), GluN2B (n = 5 each for statistical analyses), tau (n = 8 each for statistical analyses, phospho T231, 1:1000, Abcam), and actin antibodies.

Immunoblot results were quantified via a C-Digit blot scanner (LI-COR Biosciences, NE, USA) and normalized with actin or GAPDH (supplementary Fig. S7) and the expression ratios of Aβ-GFP Tg were calculated with the values of non-Tg mice as 1.

### Electrophysiological experiments

*In vitro* brain slices were prepared from 2-month-old mice (n = 6–7 animals each) as described previously^57^ with the following modifications. Briefly, mice were anaesthetized with isoflurane and pentobarbital, and then transcardially perfused with ice-cold artificial cerebrospinal fluid (aCSF), containing (in mM) 125 NaCl, 1.25 NaH_2_PO_4_, 2.5 KCl, 24 NaHCO_3_, 10 glucose, 0.5 CaCl_2_ and 8 MgSO_4_, saturated with 95%O_2_/5%CO_2_ (carbogen). Hippocampi were sliced (400 µm) and recovered in pre-warmed aCSF, which contains (in mM) 125 NaCl, 1.25 NaH_2_PO_4_, 2.5 KCl, 24 NaHCO_3_, 10 glucose, 2.5 CaCl_2_ and 1.3 MgSO_4_, 35 °C, at least 2 h before use. Transverse slices were placed on custom-made recording chamber as previously described^58^, in which both sides of slice were perfused with carbogenized aCSF at 2–3 ml/min at 28 °C. Field EPSPs (fEPSPs) were elicited by 10–100 μA constant currents pulses (100-μs duration) with a Pt bipolar electrode (FHC, ME, USA) onto the Schaffer-collateral pathway and recorded with a glass electrode filled with aCSF (1–2 MΩ). fEPSPs were amplified by multiclamp700A (Molecular Device, CA, USA) and digitized at 10 Hz (National Instruments, TX, USA) controlled by WinLTP programs^59^ (WinLTP Ltd., Bristol, UK).

To set stimulus intensities, maximum responses were determined by increasing the stimulus intensity stepwise to saturating responses. For paired-pulse and long-term potentiation (LTP) experiments, stimulus intensities that elite about 33% of the maximum response were employed. Input-output relationships were studied by plotting fiber volley response amplitude (mV) against fEPSP slope (mV/ms). Paired-pulse responses were also recorded with various inter-pulse intervals of 500 ms, 200 ms, 100 ms, 50 ms, 20 ms and paired-pulse ratios (second pulse response to first response) were calculated. LTP was induced by four theta burst stimulations, consisting of 4 bursts at 0.05 Hz of theta burst stimuli (4 bursts at 10 Hz of 5 pulse at 100 Hz).

### Behavioral analyses

Eighteen-month-old Aβ-GFP Tg and non-Tg mice were tested for open-field (OFT) and novel object recognition (NORT) (n = 11/group). All tests were carried out between 10:00 and 17:00, and all apparatuses were cleaned with sodium hypochlorite after each trial. Mice were given handling (5 min/day) for 3 days before the behavioral test.

The open-field consisted of a rectangular box (measuring area; 435 mm (W) × 275 mm (D) × 115 mm (H), SCANET, Melquest, Toyama, Japan). The apparatus was placed in an isolation cabinet that was illuminated by upper light (around 219 lux). Mice were placed into the center of the open-field arena and allowed to move freely for 10 min. The total distance moved and the total number of rearings were recorded as a measure for exploratory behavior and locomotion activity.

After the OFT, mice were habituated to the box of NORT for 5 min/day over 3 days (Fig. 8D). Each NORT consisted of three phases: (i) Sample phase; (ii) Retention interval; (iii) Test phase (Fig. 8E). During the sample phase, mice were allowed to explore two identical objects for 10 min. Subsequently, after a retention interval (1 or 24 hours), the test phase was performed. Mice were re-exposed for 10 min to one familiar object and one novel object and the exploration times to each object were recorded. Exploratory behavior was consisted of sniffing or touching the object with the nose and/or forepaws. Object recognition memory was calculated as the “preference index” (PI) = (exploration time of novel object)/(exploration time of familiar and novel objects). Based on an omission criterion of less than 30 sec exploration of objects during the test phase, we omitted several mice from the statistical analysis (non-Tg mice: n = 2–3, Aβ-GFP Tg mice: n = 2). NORT was also performed on young Aβ-GFP Tg and non-Tg mice (2–3-month-old male mice, n = 20/group each) to examine their memory function. No young mice were omitted by the omission criterion same as above.

### Statistical analyses

Statistical analyses of behavioral data were performed using SPSS software (IBM, IL, USA). Other statistical analyses comparing two samples were performed using KyPolt^TM^ 5.0 software (KyensLab Inc. Japan). Multiple analyses were conducted using GraphPad Prism 7 software (GraphPad Software, CA, USA).

## Supplementary information

Supplementary Information

## Data Availability

The datasets generated and/or analyzed during the current study are available from the corresponding author on reasonable request.
