# Supplementary material for: New Alzheimer’s disease model mouse specialized for analyzing the function and toxicity of intraneuronal Amyloid β oligomers
Source: Sci Rep. 2019 Nov 22;9:17368. doi: 10.1038/s41598-019-53415-8 (PMC6874556; doi:10.1038/s41598-019-53415-8)
Supplement: Supplementary file 1 — Supplementary Information [file 41598_2019_53415_MOESM1_ESM.pdf]

Supplementary Information

New Alzheimer's disease model mouse specialized for analyzing the function and toxicity of intraneuronal Amyloid β oligomers

Tomoyo Ochiishi, Masami Kaku, Kazuyuki Kiyosue, Motomichi Doi, Takao Urabe, Nobutaka Hattori, Hideki Shimura, Tatsuhiko Ebihara

Supplementary Methods

Immunohistochemistry

Aβ-GFP Tg and non-Tg littermates approximately 18 months of age were used. For microglia staining, coronal sections (40µm) were incubated with microglia marker Iba1(1:1000, polyclonal, FUJIFILM Wako Pure Chemical Corporation). For double labeling of 6E10 and GFP, sections were incubated with a mixture of 6E10 (1:500) and anti-GFP antibodies (1:500, chicken IgY fraction, Aves Labs Inc., OR, USA). After incubating overnight at 4°C, Iba1 and 6E10 staining were visualized with an Alexa 568-conjugated secondary antibody (1:500) and GFP staining was visualized with CF647 goat anti-chicken IgY (1:1000, Biotium Inc, CA, USA). Nuclei were labeled with DAPI. Images were taken with a BZ-X800 fluorescent microscope.

Methoxy-X04 staining

For assessment of amyloid plaques, methoxy-X04 staining was performed (Leinenga and Götz, 2018). Sections were incubated with 10 µM methoxy-X04 (Tocris Bioscience, Bristol, UK) in 50% DMSO/50% NaCl 0.9% (pH 12) for 10 min and washed twice with PBS. Then sections were mounted with fluoromount/Plus™ (Diagnostic BioSystems, CA, USA). Images were taken with a BZ-X800 fluorescent microscope.

Immunoprecipitation and immunoblot analyses

Homogenates of Aβ-GFP Tg (using a mixture of 3 animals) and GFP-Tg cortex (using a mixture of 2 animals) at 3 months of age were immunoprecipitated by GFP-Trap®_MA (Chromotek, Planegg-Martinsried, Germany) according to the manufacturer's information. Co-immunoprecipitated Aβ was detected by western blot using 6E10 antibody and ECL.

Immunoblots of synaptosome fractions were performed as described in "Methods" section. Each protein expression was normalized by GAPDH (1:2000, Abcam).

Dot blot

Homogenates of hippocampi from Aβ-GFP Tg at 18 months of age were used. Proteins (10µg/spot) were loaded on a nitrocellulose membrane. The membrane was incubated by anti-amyloid fibrils (OC) antibody (1:1000, Arigo Biolaboratories, Hsinchu, Taiwan), followed by HRP-conjugated anti-rabbit IgG and visualized by ECL.

Supplementary Figure S1

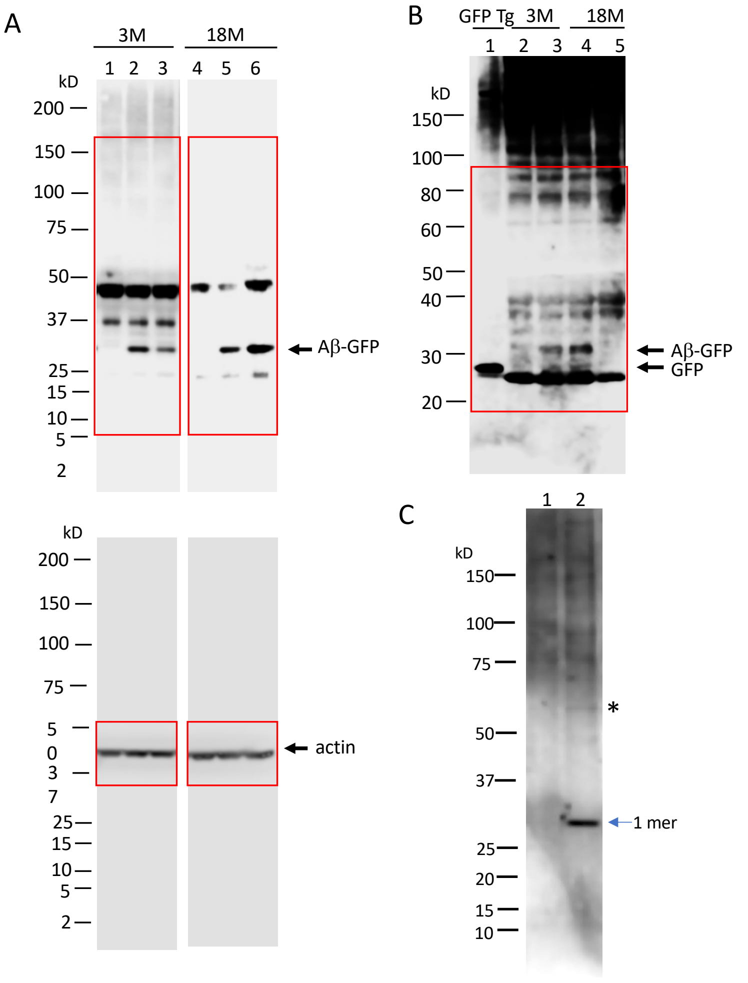

Supplementary Figure S1: Full length blots for Figure 1 and Oligomer formation of Aβ-GFP Tg mice.

A: The upper panels show the immunoblot analyses of Aβ-GFP Tg mice brain homogenate by anti-Aβ antibody. The lower panels show the immunoblot analyses by anti-actin antibody using the stripped membrane that used in each blot of upper panels.

B: The immunoblot analyses of Aβ-GFP Tg mice brain homogenate by anti-GFP antibody. Cropped areas in Fig 1A and B were showed in red squares.

C: Brain homogenates of 3-month-old Aβ-GFP Tg mice were immunoprecipitated with GFP-Trap®_MA beads and western blots were performed by 6E10 antibody. A monomer and possibly dimer (*) bands were detected. Lane1, GFP Tg mice; lane2, Aβ-GFP Tg mice.

Supplementary Figure S2

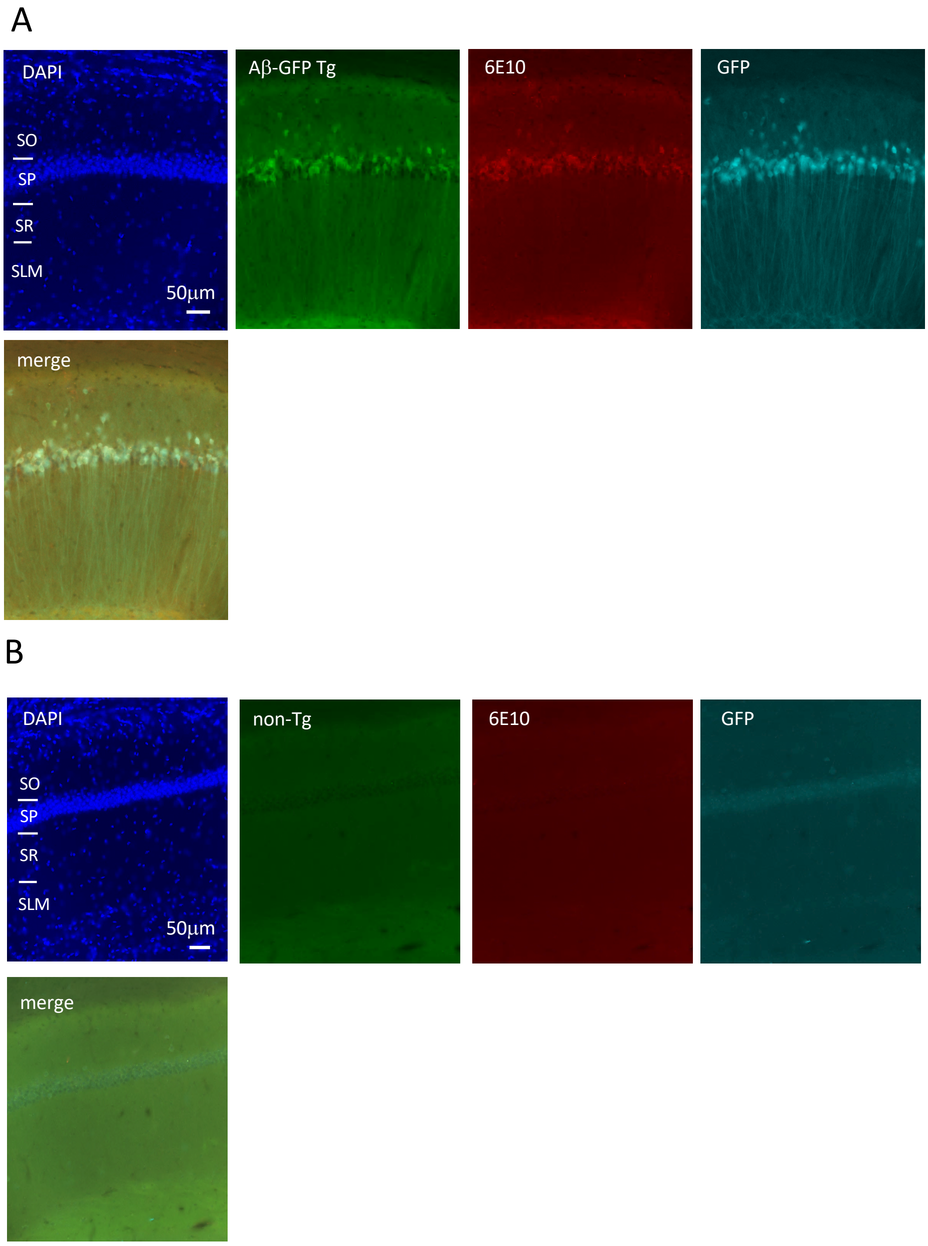

Supplementary Figure S2: Co-localization of Aβ and GFP in Aβ-GFP Tg mice. 18-month-old Aβ-GFP Tg mice and non-Tg mice were double labeled with 6E10 and GFP antibodies. Immunostained GFP was visualized by CF674 antibody. GFP and 6E10 staining co-localized in pyramidal cells of hippocampi in Aβ-GFP Tg mice (A). No immunoreactivities were detected in non-Tg mice (B). Scale bars: 50µm

Supplementary Figure S3

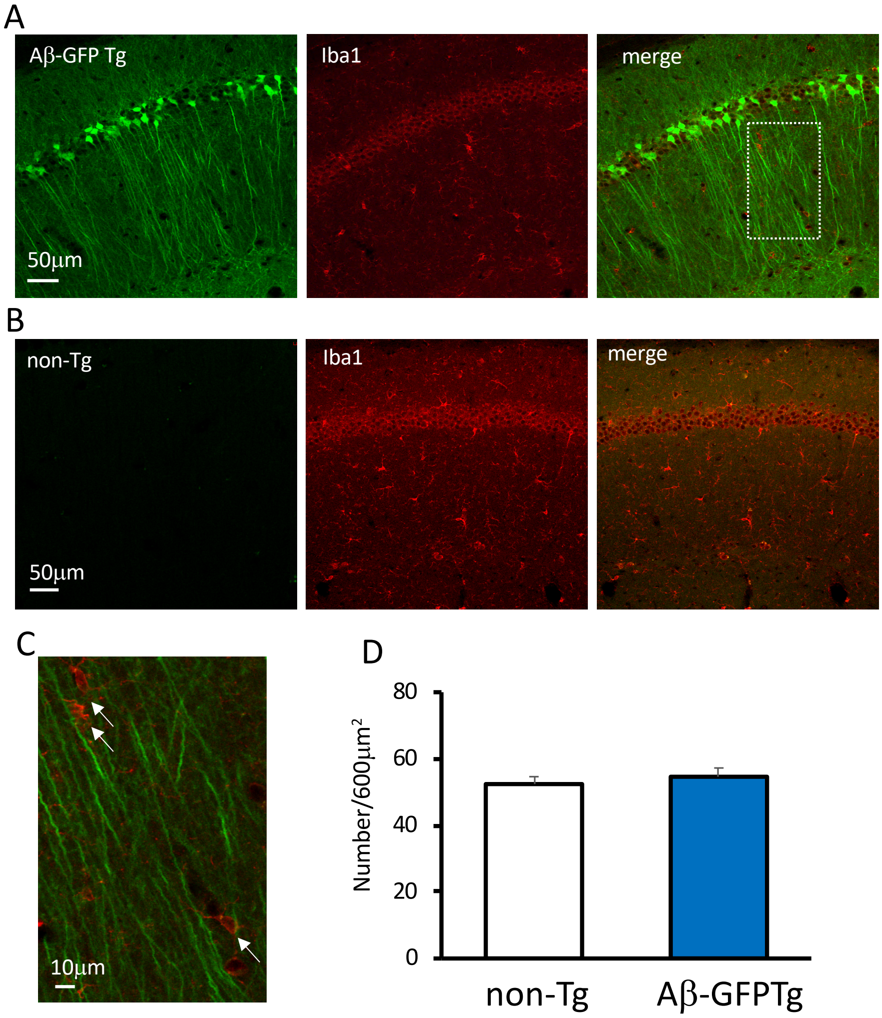

Supplementary Figure S3: Expression of Aβ-GFP fusion protein in microglia.

A-C: Hippocampi from 3-months-old Aβ-GFP Tg and non-Tg mice were immunostained with microglia marker, Iba1. Rectangle in the right panel in A was magnified in C (arrows show each microglia). As shown in the merged image in (A), Iba1 immunopositive microglia do not express Aβ-GFP fusion proteins.

D: Statistical analysis of the number of microglia per unit area of hippocampus. There was no significant difference between Aβ-GFP Tg and non-Tg mice (Mann-Whitney's U test, n=5 animals each, data are presented as means ± SEM). Scale bars; 50μm(A, B), 10μm(C).

Supplementary Figure S4

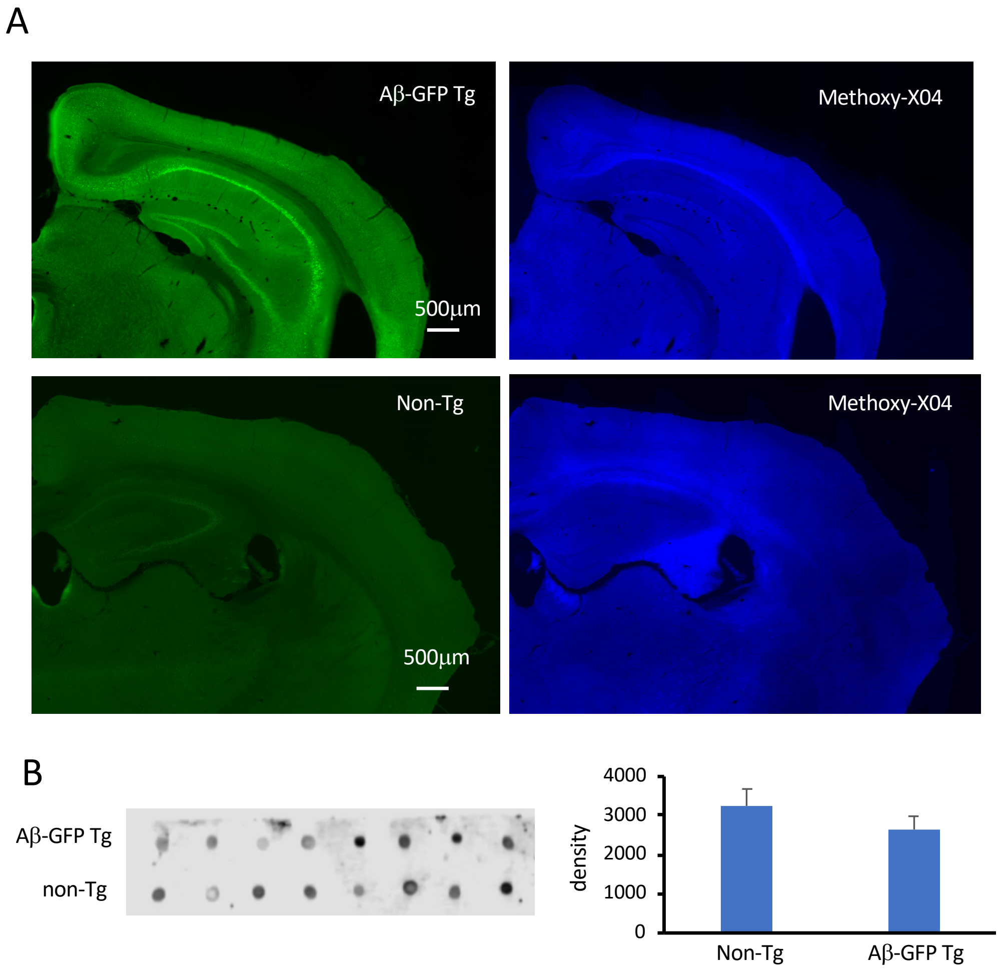

Supplementary Figure S4: Confirmation of no plaques and no extra fibril formation in 18-month-old Aβ-GFP Tg mice

A: 18-month-old Aβ-GFP Tg mice were stained by methoxy-X04 that is a fluorescent Aβ probe for the detection of plaque. No amyloid plaques were detected in any regions we examined in aged Aβ-GFP Tg mice brain, similar to non-Tg littermates.

B: Dot blot analyses by anti-amyloid fibrils antibody. The density of each spot of individual 18-month-old Aβ-GFP Tg (upper) and non-Tg (lower) mice hippocampal homogenate was measured. Histogram showed that no extra fibril formations were observed in old Aβ-GFP Tg mice compared with the non-Tg mice (Mann-Whitney's U test, n=8 animals each, data are presented as means ± SEM).

Supplementary figure S5

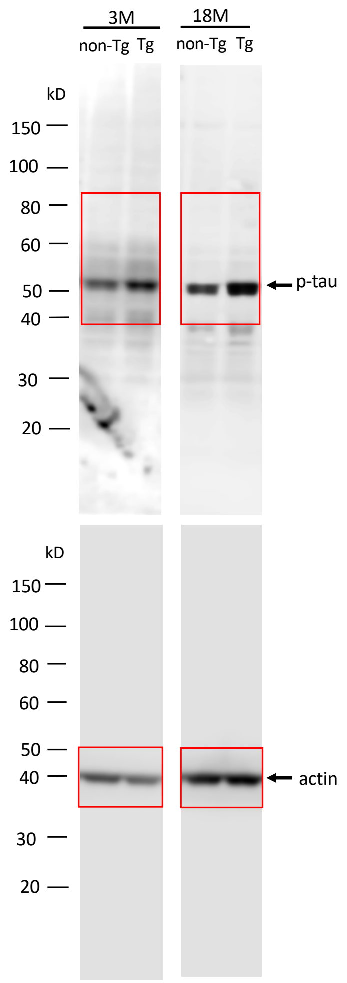

Supplementary Figure S5: Full length blots for Figure 3C.

The upper panels show the immunoblot analyses of Aβ-GFP Tg mice brain homogenate by anti-phosphorylated tau (phosphor T231) antibody. The lower panels show the immunoblot analyses by anti-actin antibody using the stripped membrane that used in each blot of upper panels. Cropped areas in Fig 3C were showed in red squares.

Supplementary figure S6

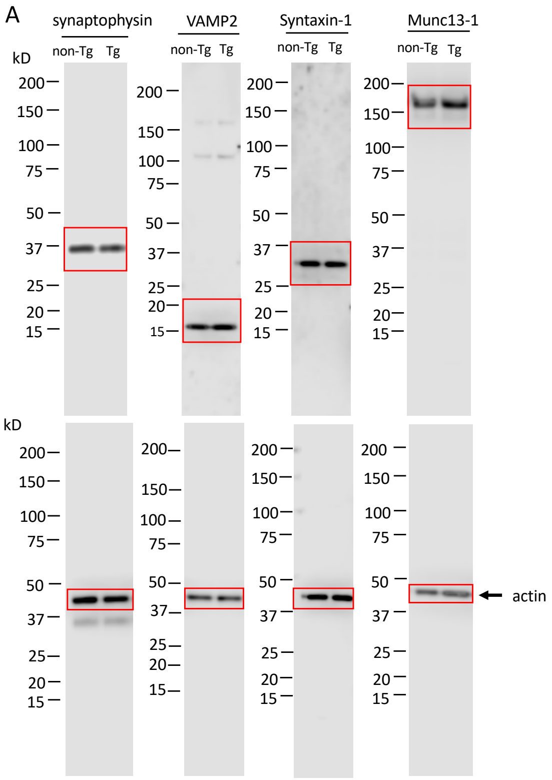

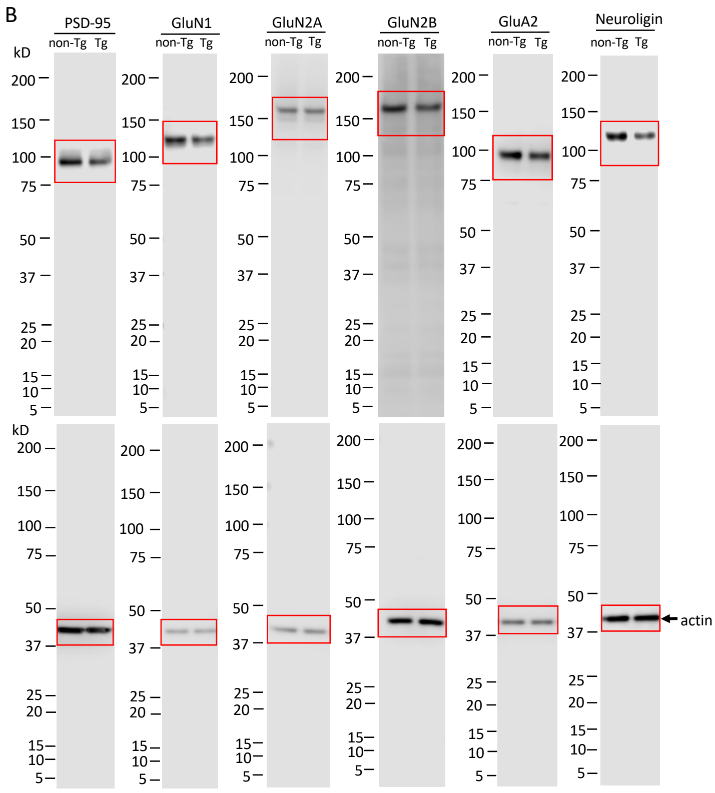

Supplementary Figure S6: Full length blots for Figure 7A and 7B. The upper panels in A and B show the immunoblot analyses of presynaptic (A) and postsynaptic (B) proteins of Aβ-GFP Tg mice. The lower panels show the immunoblot analyses by anti-actin antibody using the stripped membrane that used in each blot of upper panels. Cropped areas in Fig 7A and 7B were showed in red squares.

Supplementary figure S7

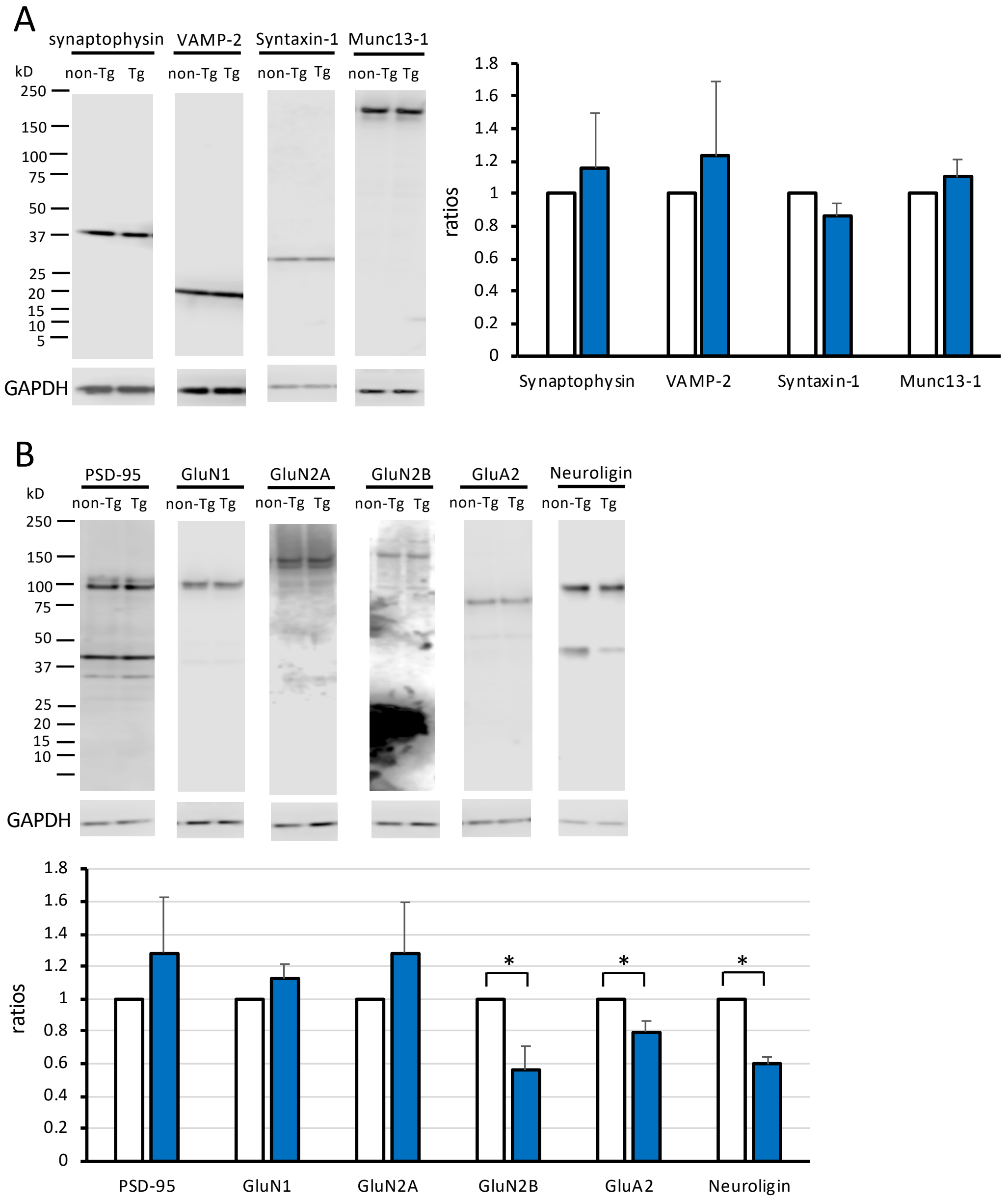

Supplementary Figure S7: Expression of the pre and post synaptic proteins in Aβ-GFP Tg and nonTg mice.

Each result was normalized by GAPDH. The results were the same as when normalized by actin.

Supplementary Reference

Leinenga, G. & Götz, J. Safety and efficacy of scanning ultrasound treatment of aged APP23 mice. *Frontiers in Neurosci.*12:55;10.3389/fnins.2018.00055 (2018)
